# 1070. Effects of two different mechanical ventilation strategies on lung conditions after experimental ARDS following blunt chest trauma and pulmonary contusion in pigs

**DOI:** 10.1186/2197-425X-2-S1-P86

**Published:** 2014-09-26

**Authors:** S Hammermüller, N Carvalho, S Huckauf, S Kobelt, M Baunack, K Noreikat, A Beda, A Reske, H Wrigge, AW Reske

**Affiliations:** Department of Anesthesiology and Intensive Care Medicine, University Leipzig, Leipzig, Germany; Department of Electronic Engineering, Federal, University of Minas Gerais, Belo Horizonte, Brazil; Fachkrankenhaus Coswig, Coswig, Germany

## Introduction

Pulmonary contusion (PC) is common after blunt chest trauma, leads to inhomogeneous lung injury and can result in acute respiratory distress syndrome (ARDS) [1]. Strategies for mechanical ventilation (MV) with different physiological rationales and approaches to positive end-expiratory pressure (PEEP) and tidal volume (TV) adjustment are proposed[1–3].

## Objective

To study the effects of the ARDSnetwork lower PEEP (ARDSnet) 1 and the Open Lung Concept (OLC) 2strategies for MV on lung ventilation and function over 24 hours in pigs after experimental PC.

## Methods

Pigs (n=16) were anesthetized, tracheotomized and received MV. Catheters were placed aseptically. Cefuroxime 750mg was given IV q6h. Unilateral PC was induced by a 10 kg weight dropped from 1.85 m height on a predefined location of the right chest. Chest tubes were inserted on both sides. Conditions comparable to an ICU were established. At 90 min after PC (post-PC) pigs were randomized to 24 hours of MV using ARDSnet (n=8) or OLC (n=8). Pressure controlled MV in the OLC group involved: an initial recruitment maneuver (50 cmH_2_O, 10 breaths), respiratory rate 80/min, I/E 2:1, TV< 6 ml/kgBW, positive inspiratory pressure (PIP) ≤30 cmH_2_O. Total PEEP of approx. 19 cmH_2_O resulted from development of intrinsic PEEP on top of external PEEP of 10 cmH_2_O. Cardiorespiratory, gas exchange and extra-vascular lung water (EVLW, single-indicator transpulmonary thermodilution) parameters were measured. Electrical impedance tomography was used to assess changes in lung ventilation (Vent). Vent was calculated as the number of pixels showing an impedance change of >15% of the global impedance change and expressed as % of baseline. Data are given as median and interquartile (25^th^-75^th^) range. Mann-Whitney-tests and General Linear Model statistics were used.

## Results

Cardiorespiratory conditions were stable without significant between-group differences at pre-PC and post-PC. At 24 hours after randomization PEEP was significantly lower in ARDSnet (8 (5-10) cmH_2_O) vs. OLC 19 (17-21) cmH_2_O). PaO_2_/FIO_2_ (477 (296-514) vs. 87 (72-118) mmHg) and static compliance were significantly higher in OLC (Tab.1). Intrapulmonary shunt (28 (27-36) vs. 11 (8-18) %), PaCO_2_ (46 (43-63) vs. 43 (33-45) mmHg), TV (7 (7-7) vs. 5 (5-6) ml/kg BW), driving pressure (deltaP) and EVLW were all significantly higher in ARDSnet after 24 hours, whereas Vent was significantly lower (Table 1). The difference in PIP (OLC 29 (27-30) cmH_2_O vs. ARDSnet 34 (29-38) cmH_2_O) was not statistically significant.

**Table 1:** Table 1

	Group	Pre-PC	Post-PC	4 hrs.	8 hrs.	12 hrs.	16 hrs.	20 hrs.	24 hrs.
Compliance (ml/cmH2O)	ARDSnet	25 (23-30)	18 (17-20)	18 (16-19)	17 (16-19)	17 (16-19)	16 (13-17)	15 (14-16)	13 (11-17)
	OLC	25 (21-34)	17 (15-18)	15 (16-19)	19 (14-23)	18 (17-23)	21 (17-26)	23 (17-26)	21 (17-24)
EVLW (ml/kg)	ARDSnet	333 (299-377)	344 (285-379)	280 (254-347)	338 (368-372)	368 (283-411)	361 (334-398)	346 (339-365)	375 (350-410)
	OLC	333 (283-345)	345 (284-411)	320 (275-378)	327 (268-365)	318 (276-326)	327 (273-372)	311 (269-359)	321 (290-397)
Ventilated area (Vent%)	ARDSnet	100	90 (72-94)	81 (77-90)	77 (73-83)	80 (76-92)	79 (76-92)	76 (71-87)	77 (72-90)
	OLC	100	90 (86-98)	93 (70-103)	94 (85-100)	98 (88-110)	99 (93-116)	99 (93-116)	104 (94-167)
deltaP (cmH2O)	ARDSnet	16 (13-22)	21 (19-23)	20 (19-23)	21 (19-25)	22 (19-25)	22 (19-26)	22 (17-28)	24 (19-28)
	OLC	21 (17-24)	26 (21-27)	9 (8-11)	10 (8-11)	10 (8-11)	10 (8-11)	10 (9-11)	10 (9-11)

## Conclusions

OLC ventilation better fulfilled common criteria for lung protection, because it facilitated MV with lower TV, deltaP, less edema (EVLW) and better lung function. It also prevented progressive derecruitment (decrease in Vent) during lung protective ventilation.
